# Supplementary figures and images for: Comparison of vaccination efficacy using live or ultraviolet-inactivated influenza viruses introduced by different routes in a mouse model
Source: PLoS One. 2022 Oct 10;17(10):e0275722. doi: 10.1371/journal.pone.0275722 (PMC9550053; doi:10.1371/journal.pone.0275722)

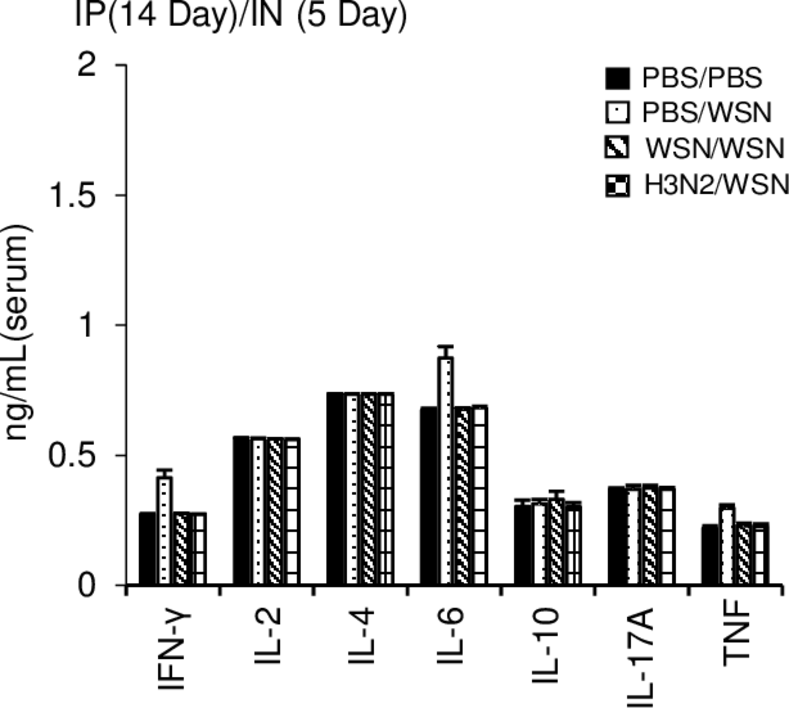

Supplement: S1 Fig — BALB/c mice (n = 5) were intraperitoneally inoculated with PBS, live WSN or live H3N2 Php. After 14 days, the mice were challenged intranasally with PBS or 10 LD50 of WSN. Sera were prepared at day 5 after the challenge, and levels of cytokines were measured by cytokine bead array. (TIF) [file pone.0275722.s001.tif]
